# Prevalence of left ventricular systolic dysfunction in a typical outpatient pacemaker cohort

**DOI:** 10.1007/s00399-020-00683-x

**Published:** 2020-05-06

**Authors:** Marius Schwerg, Henryk Dreger, Karl Stangl, Volker Leonhardt, Christoph Melzer

**Affiliations:** 1 Department of Cardiology and Angiology, Campus Mitte, Charité—Universitätsmedizin Berlin, Charitéplatz 1, 10117 Berlin, Germany; 2 Herzschrittmacher- und ICD-Zentrum Berlin, Berlin, Germany

**Keywords:** Right ventricular pacing, Transthoracic echocardiography, Screening, Heart failure, Pacemaker-induced cardiomyopathy, Rechts ventrikuläre Stimulation, Tranthorakale Echokardiographie, Herzinsuffizienz, Schrittmacher induzierte Kardiomyopathie, Screening

## Abstract

**Background:**

Right ventricular (RV) pacing is the standard treatment for symptomatic bradycardia. RV pacing is known to cause dyssyncrony. New treatment options like His bundle pacing enhance the focus on left ventricular dysfunction in patients with pacemakers.

**Objectives:**

The aim of this cross-sectional study was to obtain a real-life picture of the patients in a representative cohort of outpatients with permanent pacemakers. The prevalence and causes of left ventricular dysfunction (LVD) were explored.

**Methods:**

In total, 1869 patients of a pacemaker outpatient clinic were screened for left ventricular systolic dysfunction by transthoracic echocardiography. All patients were interviewed for symptoms and cardiologist care. Percentages of RV pacing and underlying cardiac disease were recorded.

**Results:**

A left ventricular ejection fraction (LVEF) under 45% was found in 207 (11.1%) of all patients. Predictive factors for a reduced LVEF were a high pacing rate and long-term pacing. LVD due to RV pacing was diagnosed in 3.4% of all patients. Only 845 patients (45%) reported that they regularly visited a cardiologist.

**Conclusion:**

There is a high prevalence of unknown LVD in a typical pacemaker cohort. Therefore, regular echocardiographic examinations should be performed in outpatients of pacemaker clinics.

## Abstract (de)

**Hintergrund:**

Die rechtsventrikuläre (RV) Stimulation ist die Standardbehandlung bei symptomatischer Bradykardie. Es ist bekannt, dass die RV-Stimulation eine Dyssynchronie verursacht. Neue Behandlungsoptionen wie die Stimulation des His-Bündels verstärken den Fokus auf die linksventrikuläre Dysfunktion (LVD) bei Patienten mit Herzschrittmachern.

**Fragestellung:**

Ziel dieser Querschnittsstudie war es, ein reales Bild der Patienten in einer repräsentativen Kohorte von ambulanten Patienten mit permanenten Herzschrittmachern zu erhalten. Wir untersuchten Prävalenz und Ursachen der LVD.

**Methoden:**

Insgesamt 1869 Patienten einer Schrittmacherambulanz wurden mittels transthorakaler Echokardiographie auf eine linksventrikuläre systolische Dysfunktion untersucht. Alle Patienten wurden zu Symptomen und kardiologischer Betreuung befragt. Der Prozentsatz der RV-Stimulation und der zugrunde liegenden Herzerkrankungen wurde aufgezeichnet.

**Ergebnisse:**

Bei 207 (11,1 %) aller Patienten wurde eine linksventrikuläre Ejektionsfraktion (LVEF) unter 45 % gefunden. Prädiktive Faktoren für eine reduzierte LVEF waren eine hohe Stimulationsrate und eine langfristige Stimulation. Bei 3,4 % aller Patienten wurde eine LVD aufgrund einer RV-Stimulation diagnostiziert. Nur 845 Patienten (45 %) gaben an, regelmäßig einen Kardiologen aufzusuchen.

**Schlussfolgerung:**

In einer typischen Schrittmacherkohorte besteht eine hohe Prävalenz unbekannter LVD. Daher sollten bei ambulanten Patienten von Herzschrittmacherkliniken regelmäßig echokardiographische Untersuchungen durchgeführt werden.

For patients with symptomatic bradycardia, pacemaker implantation is the only effective therapy. But right ventricular pacing is known to have deleterious effects on left ventricular function. More physiological pacing options like His bundle pacing have shown positive effects on left ventricular function compared to right ventricular pacing. This study examines the prevalence and underlying disease of left ventricular dysfunction in a typical pacemaker outpatient clinic to identify potential patients that could possibly benefit from alternative pacing options like His bundle pacing or cardiac resynchronization therapy.

## Introduction and background

Implantation of a permanent cardiac pacemaker is the only effective long-term therapy for patients with symptomatic bradycardia. In Germany, 75,000 permanent pacemakers are implanted each year [5]. Despite its undisputed clinical benefits, attention has been drawn to the negative effects associated with long-term pacing of the right ventricle (RV). RV pacing causes electrical and mechanical dyssynchrony [15, 16] similar to that of left bundle branch block. This is associated with deleterious effects on cardiac function, resulting in atrial fibrillation, heart failure and death [13, 14, 18]. In particular, RV pacing may induce ventricular dysfunction [3, 7, 9].

Previous trials identified a percentage of RV pacing of >40% as a risk factor for heart failure [10, 13]. Biventricular pacing has advantages to RV pacing, but disappointed in clinical studies [11, 17]. In contrast, His bundle pacing was shown to be superior to RV pacing in a recent study published by Abdelrahman et al. [1]. Nevertheless, there is limited data on the prevalence of ventricular dysfunction in patients with pacemakers [4, 18]. The aim of this cross-sectional study was to analyze the prevalence and causes of systolic left ventricular dysfunction (LVD) in a real-life cohort to identify patients for potential upgrade to His pacing.

## Study design and investigation methods

All patients from a large pacemaker outpatient practice in Berlin, Germany, were screened for LVD by transthoracic echocardiography in a cross-sectional study. The left ventricular ejection fraction (LVEF) was determined by Simpson’s biplane method from the apical two and four chamber views at end-systole and end-diastole by the same experienced cardiologist (GE Vivid 6). LVD was defined as an LVEF ≤45% based on the inclusion criteria of the PACE trial [2]. Duration of pacing and current pacing mode were recorded. All patients were interviewed regarding symptom burden. In patients with reduced LVEF, the patients’ cardiologists and general practitioners was contacted to find out whether the systolic dysfunction and its underlying cause were already known. In patients with unknown LVD without improvement under medication, cardiac catherization was performed. In patients with LVD not explained by other common causes of heart failure such as coronary artery disease, valvular heart disease or extensive hypertension with a percentage of RV pacing over 40%, RV pacing was considered to be the main cause of LVD.

The study conforms to the Declaration of Helsinki and was approved by the ethics committee of the Charité Universitätsmedizin Berlin. All patients provided written informed consent. This work received friendly supported from Biotronik SE & Co. KG (Berlin, Germany), through the research grant number FF035.

Statistical analyses were performed using commercially available software (Graph Pad Prism 6, GraphPad Software, San Diego, CA, USA). A *p*-value <0.05 was considered statistically significant. Fisher’s exact test was performed for dichotomous variables, and the Wilcoxon-Mann-Whitney test for ordinal factors to test for statistical significance.

## Results

In 2017, 1869 consecutive pacemaker patients were screened by transthoracic echocardiography in the authors’ pacemaker outpatient clinic. Baseline characteristics are summarized in Table 1.

**Table 1:** Baseline charactersitics

	Total	LVEF >45%	LVEF <45%	*p*-Value
*Number of patients (n)*	1869	1662	207	–
*Male, n (%)*	1051 (56%)	894 (53.8%)	157 (75.8%)	<0.001
*Age (years)*	77.7 ± 10.8	77.6 ± 10.8	78.6 ± 10.8	0.07
*System, n (%)*				0.004
AAI	45 (2.4%)	44 (2.6%)	1 (0.5%)	
DDD	1387 (74.2%)	1246 (75.0%)	141 (68.1%)	
VVI	437 (23.4)	372 (22.4%)	65 (31.4%)	
*Time since first implantation (years)*	9.6 ± 7.3	9.5 ± 7.3	10.6 ± 6.8	0.004
*Percentage of RV pacing (%)*	58.1 ± 44.1	55.8 ± 44.6	77.6 ± 34.4	<0.001
*LVEF (%)*	56.1 ± 8.3	58.3 ± 5.1	38.0 ± 6.9	NA
*Patients in cardiologist care*	845 (45.2%)	732 (44.0%)	113 (54.6%)	0.01
*Patients with LVEF <45%*	207 (11.1%)	NA	NA	NA
*Patients with percentage of RV pacing >40%*	1165 (62.3%)	995 (60.0%)	170 (82%)	<0.001
*Patients with known reduced LVEF*	NA	NA	130 (62.8%)	NA

*LVEF* left ventricular ejection fraction, *RV* right ventricular

A total of 845 (45.2%) of our patients reported regularly seeing a practicing cardiologist in addition to the pacemaker outpatient clinic.

### Predictive factors for left ventricular dysfunction

In patients with LVD, the percentage of patients with VVI pacemakers was significantly higher compared to patients without LVD (*p* = 0.004).

**Fig. 1:**
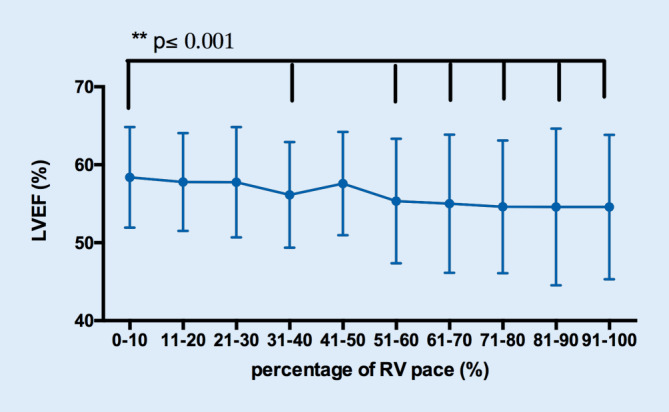
Distribution of left ventricular ejection fraction (LVEF) in relation to the proportion of right ventricular (*RV*) pacing

As shown in Fig. 1, however, a higher percentage of RV pacing correlated with a lower LVEF. A significantly reduced LVEF was already seen at a percentage of RV pacing >30%, with the lowest LVEF seen in patients with a percentage of RV pacing >90%. A reduced LVEF to below 45% was found in 11.1% of all patients. The baseline characteristics of LVD patients are given in Table 1. There was no significant age difference between LVD patients and patients with preserved left ventricular function. The percentage of males was higher in LVD patients compared to all patients (*p* < 0.001). LVD patients had a significantly longer time since implantation of the pacemaker (9.5 ± 7.3 vs. 10.6 ± 6.8 years; *p* = 0.004). The percentage of RV pacing was significantly higher in LVD patients compared with patients with preserved LVEF (56% vs. 78%: *p* < 0.0001). The proportion of patients with a percentage of RV pacing over 40% in patients with LVD was 82% in contrast to patients without LVD (OR 3.1 [2.1–4.5], *p* < 0.001).

Reduced LVEF was unknown in 37.2% of LVD patients at the time of screening. In patients without any other causes for LVD except RV pacing, reduced LVEF was unknown in 54.7% of the patients. In contrast, LVD was unknown in only 29.4% of the patients with other causes for LVD.

### Underlying disease

The underlying cause for LVD was coronary artery disease (CAD) in most cases (*n* = 71, 34%). RV pacing was assumed to be the second most common cause (*n* = 64, 31%). Other causes included, in descending order of frequency, hypertension (*n* = 25, 12%), valvular disease (*n* = 18, 9%), dilated cardiomyopathy (*n* = 12, 6%) and tachyarrhythmia (*n* = 2, 1%). In 12 cases, the cause for LVD remained unknown. In 3.4% of all patients, RV pacing alone was the suspected cause of LVD.

### Symptom burden

A total of 81% of LVD patients reported symptoms consistent with heart failure (New York Heart Association [NYHA] II–IV): 1.5% patients were in NYHA IV, 23.7% in NYHA III, 56.0% in NYHA II and 18.9% in NYHA I. In the subgroup of patients with CAD, the percentage of asymptomatic LVD was only 5.6% in contrast to the other entities of LVD with 24.2% patients in NYHA I. In the subgroup of patients with RV pacing as the reason for LVD, the percentage without symptoms of heart failure was even 31.3%.

## Discussion

With 1869 enrolled patients and a follow-up period of 9.6 years, this study is one of the largest evaluating the prevalence of LVD and symptomatic heart failure in patients with single or dual chamber pacemakers in a real world pacemaker cohort.

Considering the relevant prevalence of LVD and the finding that only 45% of all patients reported regularly seeing a cardiologist beside the pacemaker clinic, the necessity to interview pacemaker patients for symptoms of LVD at every outpatient visit is underlined.

### RV pacing and left ventricular dysfunction

Predictors for LVD were time since pacemaker implantation and high percentage of RV pacing. In contrast to previous data, LVD was already found in patients with a percentage of RV pacing over 30% and not as previously assumed with over 40%, as in the MOST trial [13]. The data in the present study support a lower threshold for the percentage of RV pacing for developing LVD [4].

### Percentage of RV pacing

The percentage of RV pacing in this study was smaller than in the MOST trial published in 2003 on patients with sinus nodal disease. However, it was similar to the study of Abdelrahman about the benefits of His bundle pacing [1]. This hopefully raises awareness for the need to prevent unnecessary RV pacing and the advances in programming. But newer studies show that new pacemaker algorithms to minimize RV pacing, pacing with long fixed atrioventricular delay and pacing with lower pacing rates for VVI pacemakers might reduce RV pacing even more to minimize the risk of LVD [6, 8, 12].

### Screening for left ventricular dysfunction

In this cohort, CAD was the most frequent cause of LVD. Interestingly, the second most common cause was a high RV pacing percentage. This emphasizes the need to minimize RV pacing in pacemaker patients. In the authors’ opinion, regular echocardiographic screening of high-risk patients with a pacing rate over 30% is justified to improve rates of diagnosis and treatment of heart failure especially due to the high portion of patients in NYHA class I among patients with LVD due to RV pacing.

### Limitations

In contrast to other reports, this study presents real life data from a regular outpatient clinic. A major limitation of the study worth noting is the lack of reliable LVEF data before pacemaker implantation. Therefore, in a proportion of patients, impaired LVEF might have already been present before pacemaker implantation.

### Outlook

Prophylactic cardiac resynchronization therapy implantation in patients with atrioventricular block and probable high pacing rates failed to improve outcome by preventing LVD [11, 17]. This might be explained by the small prevalence of LVD in our real-life cohort. Further studies need to find predictors for the development of LVD and whether other special lead locations like His bundle pacing might help to prevent this [1].

## Conclusions

The authors conclude that LVD is a common comorbidity in patients with pacemakers and, therefore, a regular echocardiographic examination is recommended. A percentage of RV pacing above 30% increases the risk of LVD in patients with pacemakers. Patients with LVD due to RV pacing are often asymptomatic.

## Practical conclusions

- Reduced LVEF of under 45% was found in 11.1% of all pacemaker patients
- Predictors of LVD was time since pacemaker implantation and percentage of RV pacing
- An increased proportion of LVD was found in patients with a percentage of RV pacing over 30%
- Only 45.2% of patients see a cardiologist regularly besides the pacemaker outpatient clinic
- LVD due to RV pacing is often asymptomatic and therefore difficult to diagnose with the normal pacemaker routine
